# Anatomico-radiological Study of the Bifurcate Ligament of the Foot with Clinical Significance

**DOI:** 10.7759/cureus.3847

**Published:** 2019-01-08

**Authors:** Rene M Kafka, Ian L Aveytua, Paul J Choi, Anthony C DiLandro, R. Shane Tubbs, Marios Loukas, Douglas N Mintz, Ritwik Baidya, Sushil Kumar, Santosh K Sangari, Estomih P Mtui, Anthony V D'Antoni

**Affiliations:** 1 Podiatry, Eastern Colorado Health Care System, Denver, USA; 2 Podiatry, Memorial Health System, Marietta, USA; 3 Surgery, Seattle Science Foundation, Seattle, USA; 4 Podiatry, New York College of Podiatric Medicine, New York, USA; 5 Neurosurgery, Seattle Science Foundation, Seattle, USA; 6 Anatomy, St. George's University, St. George, GRD; 7 Radiology, Hospital for Special Surgery, New York, USA; 8 Radiology, Weill Cornell Medical College, New York, USA

**Keywords:** anatomy, ankle sprain, bifurcate ligament, calcaneocuboid ligament, calcaneonavicular ligament, foot, foot joints, orthopedic surgery, podiatry, radiology

## Abstract

Introduction

Lateral ankle sprain caused by forcible plantar flexion and inversion of the foot commonly damages the anterior talofibular ligament and other ligaments. Unfortunately, involvement of the bifurcate ligament (BL) is often overlooked when assessing such injuries in clinical practice and identification of this ligament on magnetic resonance (MR) scans can be challenging. Anatomically, the BL is a Y-shaped structure with two bands: the calcaneonavicular ligament (CNL) and calcaneocuboid ligament (CCL). There are few anatomical studies on the morphometric characteristics of the BL and even fewer biomechanical studies. Therefore, the objective of this anatomico-radiological study was to investigate the morphology of the BL using a multifaceted approach, and classify the fiber characteristics of the CNL and CCL.

Materials and methods

We measured the length and the width of 53 embalmed cadaveric feet. Meticulous dissection of each foot was performed to expose the BL. Measurements of the length, width, thickness, and shape of the CNL and CCL were taken using a digital caliper. We also documented the fiber orientation of each ligament, and used a goniometer to measure the bifurcation angle between the CNL and CCL via two methods. Confirmatory histologic analysis of the ligaments was performed and digital radiographs of the ligaments with attached radiopaque monofilament were taken. We also included an MR scan of the BL. Using descriptive and inferential statistics, we documented any significant relationships between the variables.

Results

Mean (range) age at death of cadavers was 76 (42-94) years. The CNL was found in all the feet and the CCL was not present in 9.4% of the feet. Mean (standard deviation) length of the CNL and CCL was 22.7 (4.12) mm and 10.9 (2.53) mm, respectively. Mean (standard deviation) thickness of the CNL and CCL was 3.23 (1.56) mm and 1.48 (0.71) mm, respectively. Related to ligament morphology, the CNL was most frequently cord shaped (67.92%) and the CCL was most frequently flat shaped (83.33%). The mean bifurcation angle measured 32.75^o ^and 29.31^o ^in methods 1 and 2, respectively. The correlation between the two measured angles was very strong (*p* < 0.001).

Discussion

We found that 90.6% of feet had both the CNL and CCL, 9.4% had the CNL and no CCL, and none (0%) had the CCL and no CNL. These frequencies are similar to a recent Japanese study. Our sample of donors were American and predominantly white. Whether the difference in frequencies between the studies is related to ethnicity is unknown and requires future investigation. Interestingly, on average the CNLs were twice as long and twice as thick as the CCLs. The CCLs tended to be wider distally and tapered compared to the CNLs.

Conclusions

Our findings better classify the morphology and fiber orientation of the BL. Coupled with the radiographs and MR scan, our data may be of particular value to radiologists and surgeons. Our BL fiber orientation classification system and angle measurements can pave the way for future biomechanical studies to investigate any relationships between fiber type, angle, and strength of the constituent bands. More accurate descriptions of the BL should lead to improved diagnosis and treatment of ligamentous injuries of the foot.

## Introduction

The bifurcate ligament (BL) of the foot, also called Chopart ligament, is a strong Y-shaped structure that stabilizes the calcaneocuboid joint [1]. The BL also stabilizes the talocalcaneonavicular and midtarsal joints, and therefore, has been described as the keystone of the transverse tarsal joint [2]. The ligament is anatomically found on the dorsal surface of the calcaneocuboid joint and has a main proximal stem that attaches to the calcaneal sulcus, which then divides distally to attach to the navicular and dorsomedial aspect of the cuboid [1,3-5]. Consequently, the BL has two constituent bands: the calcaneonavicular ligament (CNL) and calcaneocuboid ligament (CCL) [1,6].

Damage to the BL may be unrecognized by clinicians in patients with inversion injuries of the feet [6-7]. The rarity of isolated BL rupture is attributed to its high mechanical stability and strength, and possibly to its relationship with the surrounding rigid tarsal framework [8]. Some authors have suggested that the BL has a mechanical coupling relationship with the talonavicular joint [3]. There have been reports of isolated bilateral dislocations of the calcaneocuboid joints that required surgical repair [9-10]. Only a few cadaveric studies have investigated the morphometry of the BL. Using embalmed Japanese cadavers, Edama et al. [11] measured the BL and reported the CNL to be 20.8 ± 2.9 mm long, 4.9 ± 1.2 mm wide, and 3.8 ± 1.1 mm thick; whereas, the CCL was approximately 10.5 ± 2.7 mm long, 4.7 ± 2.4 mm wide, and 1.5 ± 0.6 mm thick. Unfortunately, the authors did not correlate their data with diagnostic images [11]. To the best of our knowledge, no study has classified the fiber orientation of the BL and correlated its morphometry with diagnostic images.

## Materials and methods

In this study, we used our previously published methods for investigating the morphology and morphometry of human ligaments [12-16] and followed evidence-based guidelines for reporting original anatomical studies [17]. After measuring the length and width of 53 non-dissected, embalmed feet from 33 adult cadavers, we performed meticulous dissections of each foot to expose the BL. We measured and recorded the length, width, thickness, and shape of the CNL and CCL using a digital caliper (Hawk Inc., Cleveland, OH). For ligament width, we measured overall, proximal, middle and distal widths because of the striking morphologic changes that were observed as the ligaments coursed distally to attach to their respective bones. We also recorded the fiber orientation of each ligament, and measured the bifurcation angle (using two methods) between the CNL and CCL with a goniometer (Prestige Medical, Northridge, CA).  Histologic analysis of the BLs was performed to ensure the measured tissues were ligaments. Digital radiographs of the ligaments with attached radiopaque monofilament were taken using a model 715 A-BD X-ray unit (X-CEL X-ray Inc., Crystal Lake, IL). Additionally, a sagittal magnetic resonance (MR) scan from the left foot of a 30-year-old man was included to show how the BL looks on MR imaging.

Data are presented as means (standard deviations, SDs), ranges, or percentages, whenever appropriate. Descriptive and inferential statistics were used to analyze the data and analyses performed using IBM SPSS Statistics Version 20 (IBM, Armonk, NY). A *p*-value of less than 0.05 was considered to be statistically significant.

## Results

We measured the length and width of 53 embalmed feet from 33 adult cadavers (24 females and nine males) with a mean (range) age at death of 76 (42-94) years (Table 1).

**Table 1:** Demographics of sample and foot measurements ^a^ Only one lower limb was available for twelve females. ^b^ Only one lower limb was available for one male. ^c^ Length of foot measured from posterior aspect of calcaneal tubercle to distal end of second toe. ^d^ Width of foot measured from lateral aspect of fifth metatarsal head to medial aspect of first metatarsal head.

Characteristics	Females (n=24)	Males (n=9)
Right foot (no.)	18 ^a^	8 ^b^
Left foot (no.)	18 ^a^	9 ^b^
Mean (range) age at death (years)	78 (53-94)	70 (42-91)
Mean length of foot (cm) ^c^	20.91	23.12
Mean width of foot (cm) ^d^	7.54	8.25

Frequency of the CNL and CCL

There were 26 right feet and 27 left feet (total of 53 feet). The CNL was present in all cases (100%) and the CCL in 48 out of 53 feet (90.6%). In one foot, there was gross evidence of CNL ossification and in another the CCL was torn.

Morphology and morphometry of the CNL and CCL

**Figure 1:**
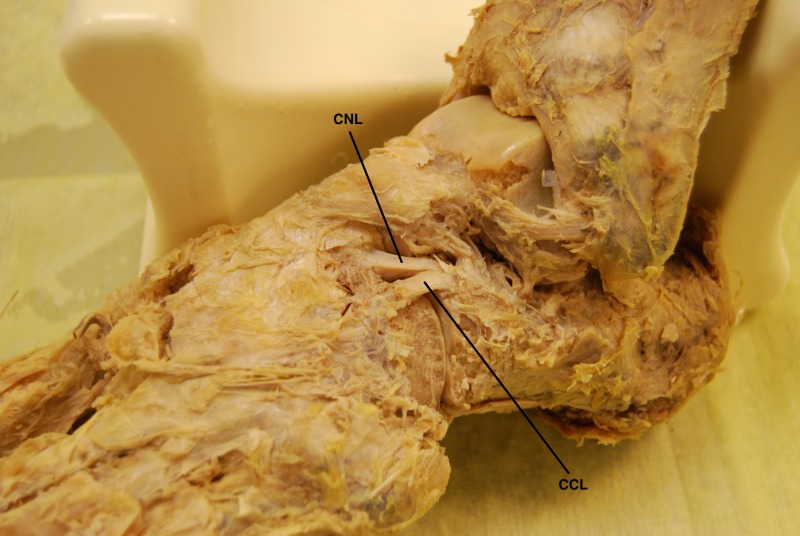
Superolateral view of the bifurcate ligament (BL) and its constituent bands in a cadaveric left foot The bifurcate ligament is demonstrated in the center of the photograph. Note how the calcaneonavicular ligament (CNL) is cord shaped and the calcaneocuboid ligament (CCL) is flat shaped in this dissected foot.

**Figure 2:**
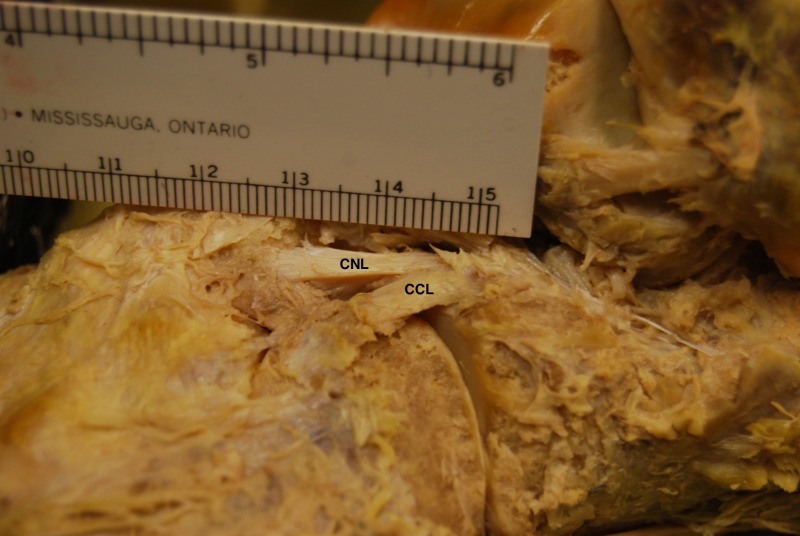
Superolateral view of the bifurcate ligament (BL) and its constituent bands in a cadaveric left foot Magnified area of Figure 1 highlighting the bifurcate ligament. Note how the calcaneonavicular ligament (CNL) is cord shaped and the calcaneocuboid ligament (CCL) is flat shaped. This was the most common arrangement found in our sample of cadaveric feet.

**Figure 3:**
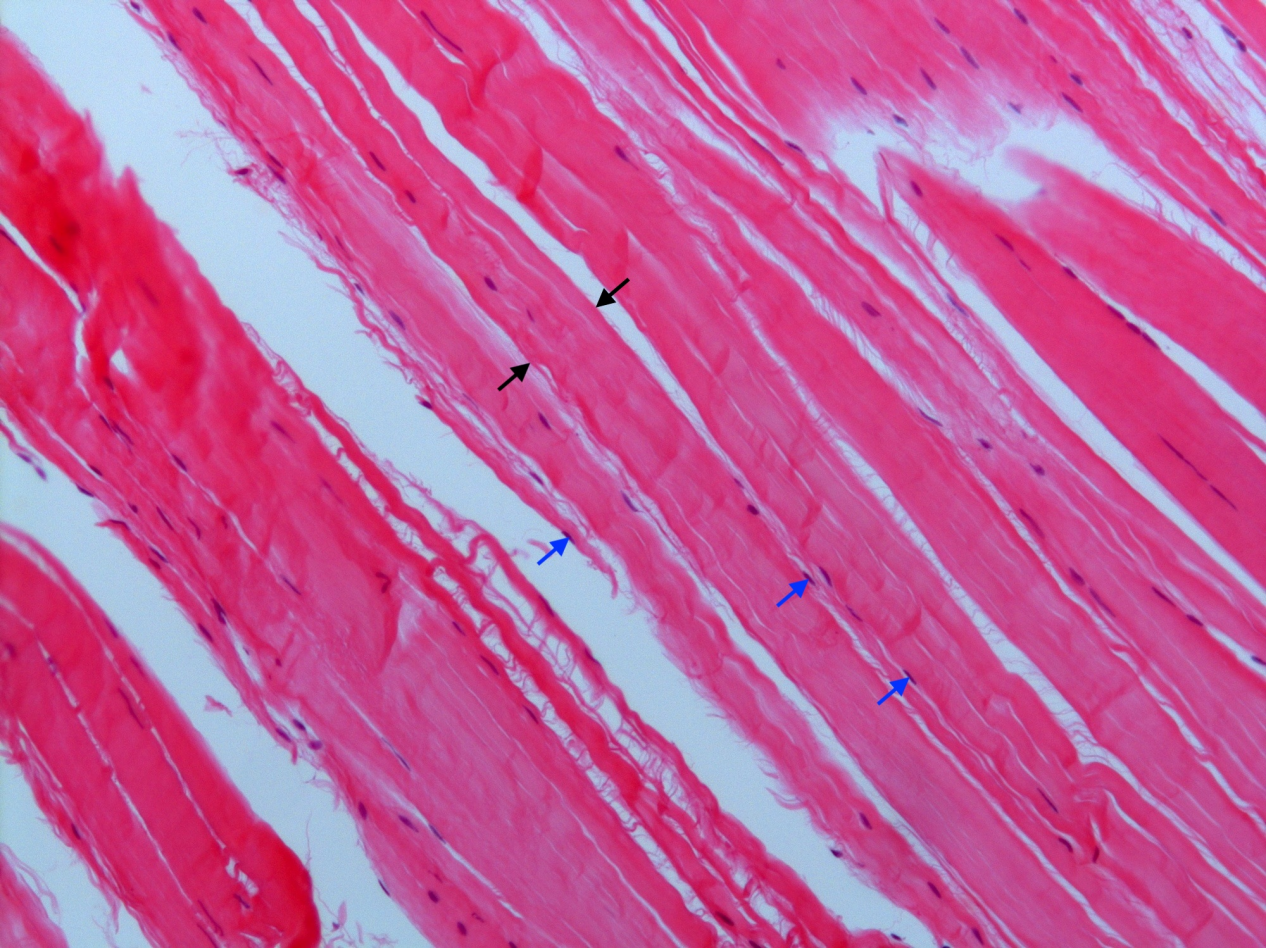
Histology of the calcaneocuboid ligament Confirmatory histologic analysis of both ligamentous bands was performed, although only the calcaneocuboid ligament is shown in this photomicrograph. Both bands demonstrated dense regular connective tissue with interposed fibroblasts, characteristic of ligaments. The black arrows delimit a thick band of collagen fibers. The tips of the blue arrows point to the nuclei of fibroblasts (Haematoxylin and eosin stain x100).

While the CNL was cord shaped in the majority of feet (36 of 53 feet, 67.92%), the CCL was most frequently flat shaped (40 of 48 feet, 83.33%). On average, the CNLs were longer (*p* < 0.05, paired t test) and thicker (*p* < 0.05, Wilcoxon signed-rank test) than the CCLs. Table 2 shows the morphometric characteristics of the BL and contains the mean (SD) length, thickness, and width (overall and at their proximal, middle and distal portions) of both ligaments. Overall mean width between both ligaments was not significant (*p* > 0.05, paired t test). The CNL is usually more cord shaped, and the comparative morphologies of both ligaments can be observed in Figures 1-2. Histologic analysis was done on both bands to confirm that they were ligaments (Figure 3).

**Table 2:** Morphometric characteristics of the bifurcate ligament NB: All data are shown in millimeters. ^a ^The calcaneocuboid ligament was absent in five feet (n=48). ^b ^Note that the calcaneocuboid ligament was torn at its midportion in one foot (n=47). ^c^ *p*-value < 0.05 is significant. ^d ^Mean overall width was calculated by taking the mean of the proximal, middle, and distal width data.

Characteristics	Calcaneonavicular ligament (n=53)	Calcaneocuboid ligament (n=48) ^a,b^	p-value
Mean (SD) length	22.7 (4.12)	10.9 (2.53)	< 0.01 ^c^
Mean (SD) thickness	3.23 (1.56)	1.48 (0.71)	< 0.01 ^c^
Mean (SD) overall width ^d^	7.12 (1.65)	7.11 (2.51) ^b^	0.8276
Mean (SD) proximal width	9.70 (3.01)	8.19 (2.87)	0.0057 ^c^
Mean (SD) middle width	5.02 (1.23)	6.39 (2.65) ^b^	0.0033 ^c^
Mean (SD) distal width	6.64 (2.12)	6.82 (2.33)	0.9271

Male cadavers had longer and wider feet, and longer CCLs than female cadavers. Regarding the association between length of the CCL and sex, CCL length remained significant even after adjusting for the effect of foot length (*p* = 0.07), but the size of the effect was reduced, indicating that the longer CCL in males is partially attributable to their longer feet but the difference persists even when this factor is accounted for. However, longer feet had longer CCLs (Pearson *r* = 0.5209, *p* = 0.0001). No significant associations were found between mean foot width and mean width of either the CNL or CCL (Pearson *r* = 0.0151 and 0.1016, *p *= 0.9147 and 0.4966, respectively).

Angle of bifurcation

**Figure 4:**
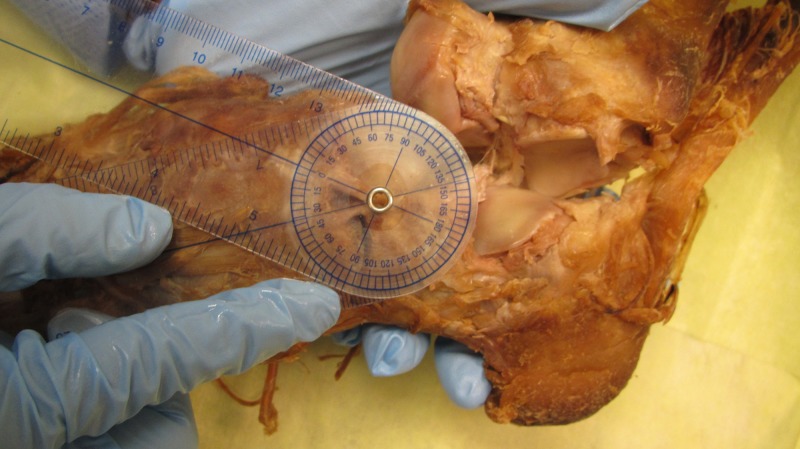
Method to identify bifurcation angle of bifurcate ligament (method two) A transparent goniometer was placed over the bifurcate ligament and the associated lines of the instrument were superimposed over the calcaneonavicular ligament (CNL) and calcaneocuboid ligament (CCL). The examiner is stabilizing the midfoot bones in place while taking the measurement. The angle between both ligaments was then recorded and is 36^o^ in this foot.

The angle between the CNL and CCL was measured by two methods using a transparent goniometer (Figure 4). For both methods, we attempted to place the reference lines of the goniometer in the center of the constituent ligaments. Method one was done by simply measuring the angle between the CNL and CCL. Method two was similar to the first method, but included manual re-approximation of the bones to compensate for possible alterations as a consequence of the dissection. The mean bifurcation angle measured 32.75^o ^and 29.31^o ^in methods one and two, respectively. The correlation between the two measurements was very strong (*p* < 0.001). There were no significant relationships between the bifurcation angle and the mean length and mean overall width of the CNL or CCL (*p* > 0.1).

Fiber orientation classification system

**Figure 5:**
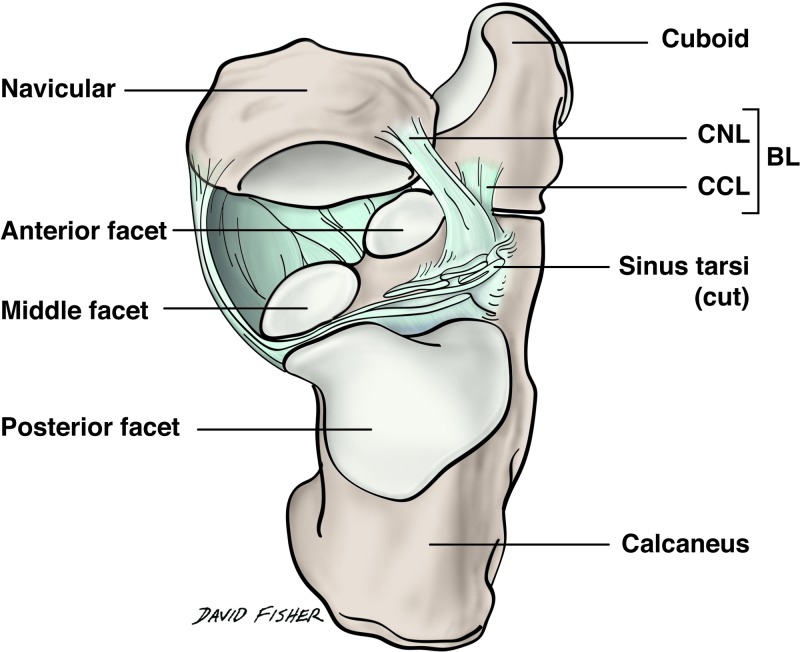
Superior view of the bifurcate ligament and its relation to the sinus tarsi The proximal calcaneonavicular and calcaneocuboid ligaments were most often blended and contiguous with the ligaments of the sinus tarsi. In this illustration, the talus has been removed so that the origin of both bands can be appreciated relative to the sinus tarsi. This arrangement was observed on most of the feet dissected in this study. BL: bifurcate ligament; CNL: calcaneonavicular ligament; CCL: calcaneocuboid ligament.

**Figure 6:**
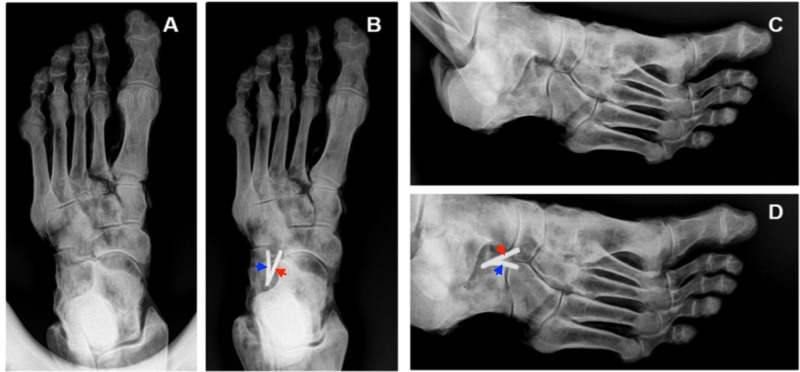
Plain film radiographs of a cadaveric left foot with and without monofilament attached to the bifurcate ligament Panel A, dorsoplantar radiograph without monofilament; Panel B, dorsoplantar radiograph with monofilament attached to the BL; Panel C, medial oblique radiograph without monofilament; Panel D, medial oblique radiograph with monofilament attached to the bifurcate ligament. In Panels B and D, note how the proximal stem of the bifurcate ligament attaches to the dorso-distal surface of the calcaneus. The most distal aspect of the calcaneonavicular ligament (red arrows) attaches to the proximo-lateral surface of the navicular, whereas the most distal aspect of the calcaneocuboid ligament (blue arrows) attaches to the proximo-medial surface of the cuboid.

**Figure 7:**
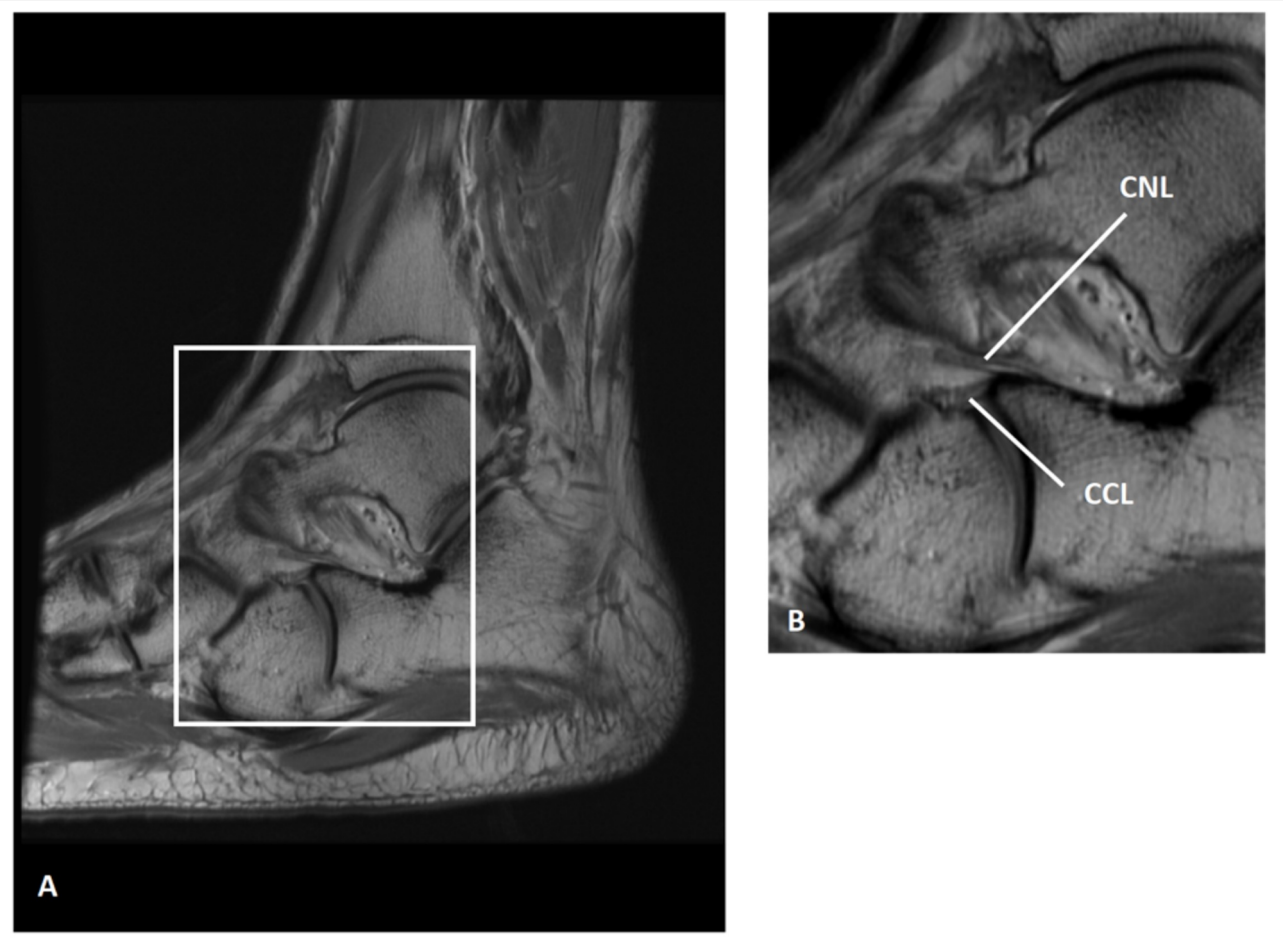
Bifurcate ligament of a 30-year-old man shown on a sagittal, proton-density magnetic resonance imaging Panel A, left foot with box delimiting the bifurcate ligament; Panel B, magnified view of the box shown in Panel A. (Images courtesy of Douglas N. Mintz, MD, FACR, Hospital for Special Surgery, NY) CNL: calcaneonavicular ligament; CCL: calcaneocuboid ligament.

Based on our data, we propose a classification system for BL shape and fiber orientation (Table 3). Both ligaments were predominantly cord-shaped or flat-shaped, and their constituent fibers were arranged in one of the following patterns: spiral, parallel, or fanned. The CNL was usually cord-shaped (36 of 53 feet, 67.92%) and the CCL was usually flat-shaped (40 of 48 feet, 83.33%). Interestingly, the proximal attachments of both the CNL and CCL on the calcaneus were most often blended with each other and with the ligaments of the sinus tarsi as shown in Figure 5. Such an arrangement is not fully appreciated in modern anatomy textbooks and atlases, and can be difficult to observe on radiographs and MR scans (Figures 6-7).

**Table 3:** Classification system of bifurcate ligament shape and fiber orientation

	Calcaneonavicular Ligament (n=53)		Calcaneocuboid Ligament (n=48)	
Morphology	Frequency	Percent (%)	Frequency	Percent (%)
Cord, parallel fibers	24	45.28	8	16.67
Cord, spiral fibers	12	22.64	0	0
Flat, parallel fibers	15	28.30	29	60.42
Flat, fanned fibers	2	3.77	11	22.92

## Discussion

In this study, we found that 90.6% of feet had both the CNL and CCL, 9.4% had a CNL and no CCL, and none (0%) had a CCL and no CNL. Our data are similar to a recent Japanese study of 100 feet that reported 68% contained both bands (type I), 32% had a CNL and no CCL (type II), and none (0%) had a CCL and no CNL [11]. Our sample of cadavers was American and predominantly white. Whether the differences in frequency between the studies is related to ethnicity is unknown and requires future investigation.

The CNL was usually cord shaped (36 of 53 feet) and the CCL usually flat shaped (40 of 48 feet). Interestingly, on average the CNLs were twice as long and twice as thick as the CCLs. The CCLs tended to be wider distally and tapered compared to the CNLs. When comparing mean length and width of the CNL and CCL relative to right and left feet, none of the variables were significant between feet except for mean CNL middle width. Mean (SD) CNL middle width was 4.71 (1.24) for left feet and 5.34 (1.16) for right feet (*p* < 0.008, paired t test). Whether this difference is correlated to handedness (right-hand versus left-hand dominant) is unknown because we did not have access to the cadavers’ medical records.

The BL has been described as a Y-shaped structure [1]. The mean angle between the CNL and CCL has been reported to be 30^o ^[8]. We found the mean bifurcation angles to be 32.75^o ^and 29.31^o ^in methods one and two, respectively. Kelikian stated that the CNL is usually stronger than the CCL, and that the CCL can be absent. Absence of the CCL was found in 9.4% of feet in the present study. Kelikian reported the mean width of the CNL at its proximal attachment to be 10 mm and we found the mean (SD) proximal width to be 9.70 (3.01) mm. Kelikian also reported the mean length of the CNL to be 20 to 25 mm and we found the mean (SD) length to be 22.7 (4.12) mm. Regarding the CCL, Kelikian reported that its width and length is approximately 5 mm and 10 mm, respectively [8]. Similarly, we found that the CCL mean (SD) overall width and length is 7.11 (2.51) mm and 10.9 (2.53) mm, respectively. Interestingly, Barclay-Smith described two parts of the CNL: a short, inferior part and an upper, more superficial part. The upper part, the main component of the ligament, is longer and stronger than the inferior part [18].

The BL is an important ligament found within the tarsal sinus [7]. Although there are few anatomical and biomechanical papers on the BL, some authors describe it as a major supporter of the calcaneocuboid joint along its dorsolateral surface [3]. There are published reports of avulsion fractures at the anterior process of the calcaneus at the site of origin of the BL [19-20].

## Conclusions

Mechanical instability of the calcaneocuboid joint with possible avulsion fracture of the anterior process of the calcaneus due to forcible inversion and plantar flexion of the foot necessitates morphometric analysis of the BL. Since more accurate descriptions of the BL and its constituent bands can lead to improved diagnosis and treatment of common foot injuries, further studies are needed to better investigate and categorize the orientation of BL fibers and its role in biomechanical stability of the foot. In this study, we used dissection in conjunction with diagnostic images (radiographs and MR scan) to better investigate the morphology and arrangement of the constituent bands of the BL. MR scans in multiple planes are necessary to appreciate the complete morphology of the BL, especially as it relates to the sinus tarsi. Our data will inevitably help radiologists assess ligament damage using computed tomography (CT) and magnetic resonance imaging (MRI) technology. Since the BL provides major support for the calcaneocuboid joint, it may be involved in the congenital foot anomaly, talipes equinovarus (clubfoot). Therefore, our morphometric and fiber-orientation data can also help orthopaedic surgeons treat clubfoot and other conditions of the foot.
